# Development and validation of anthropometric-based fat-mass prediction equations using air displacement plethysmography in Mexican infants

**DOI:** 10.1038/s41430-023-01285-9

**Published:** 2023-04-13

**Authors:** Ameyalli M. Rodríguez-Cano, Omar Piña-Ramírez, Carolina Rodríguez-Hernández, Jennifer Mier-Cabrera, Gicela Villalobos-Alcazar, Guadalupe Estrada-Gutierrez, Arturo Cardona-Pérez, Alejandra Coronado-Zarco, Otilia Perichart-Perera

**Affiliations:** 1 Nutrition and Bioprogramming Coordination, Instituto Nacional de Perinatología Isidro Espinosa de los Reyes, CP 11000 Ciudad de México, México; 2 Bioinformatics and Statistical Analysis Department, Instituto Nacional de Perinatología Isidro Espinosa de los Reyes, CP 11000 Ciudad de México, México; 3 Neonatal Ward, Instituto Nacional de Perinatología Isidro Espinosa de los Reyes, CP 11000 Ciudad de México, México; 4 Research Division, Instituto Nacional de Perinatología Isidro Espinosa de los Reyes, CP 11000 Ciudad de México, México; 5 General Director, Instituto Nacional de Perinatología Isidro Espinosa de los Reyes, CP 11000 Ciudad de México, México; 6 Neonatology Division, Instituto Nacional de Perinatología Isidro Espinosa de los Reyes, CP 11000 Ciudad de México, México

**Keywords:** Epidemiology, Nutrition, Paediatrics, Public health

## Abstract

**Background/Objectives:**

Fat-mass (FM) assessment since birth using valid methodologies is crucial since excessive adiposity represents a risk factor for adverse metabolic outcomes. Aim: To develop infant FM prediction equations using anthropometry and validate them against air-displacement plethysmography (ADP).

**Subjects/Methods:**

Clinical, anthropometric (weight, length, body-mass index –BMI–, circumferences, and skinfolds), and FM (ADP) data were collected from healthy-term infants at 1 (*n* = 133), 3 (*n* = 105), and 6 (*n* = 101) months enrolled in the OBESO perinatal cohort (Mexico City). FM prediction models were developed in 3 steps: 1) Variable Selection (LASSO regression), 2) Model behavior evaluation (12-fold cross-validation, using Theil-Sen regressions), and 3) Final model evaluation (Bland-Altman plots, Deming regression).

**Results:**

Relevant variables in the FM prediction models included BMI, circumferences (waist, thigh, and calf), and skinfolds (waist, triceps, subscapular, thigh, and calf). The R^2^ of each model was 1 M: 0.54, 3 M: 0.69, 6 M: 0.63. Predicted FM showed high correlation values (*r* ≥ 0.73, *p* < 0.001) with FM measured with ADP. There were no significant differences between predicted vs measured FM (1 M: 0.62 vs 0.6; 3 M: 1.2 vs 1.35; 6 M: 1.65 vs 1.76 kg; *p* > 0.05). Bias were: 1 M −0.021 (95%CI: −0.050 to 0.008), 3 M: 0.014 (95%CI: 0.090–0.195), 6 M: 0.108 (95%CI: 0.046–0.169).

**Conclusion:**

Anthropometry-based prediction equations are inexpensive and represent a more accessible method to estimate body composition. The proposed equations are useful for evaluating FM in Mexican infants.

## Introduction

Body composition in early infancy may play a central role in programming metabolic diseases later in life [1, 2]. Accurate fat mass (FM) measurement is important from birth and throughout life [3, 4]. At present, information on body composition is still needed, and there is no consensus about the optimal FM percentage (%FM) in infants. Infants with rapid FM accretion during their first six months of life are likelier to remain with higher FM at four years old [5]. It is essential to have age- and ethnic-specific data [3, 6, 7], especially during early infancy, where rapid growth with a wide inter-individual variability occurs [3, 8]. There is conflicting evidence about differences in FM between females and males in infancy; some authors have reported differences at birth [9, 10], while others have not [11, 12]. Biologically, females have higher FM, but this difference could emerge around 5 months of age [13].

Air displacement plethysmography (ADP) is a valid body composition assessment method that estimates FM [14–17]. However, ADP is expensive, not widely accessible, and is mainly used in research studies. It is not practical for clinical assessment or population purposes, where body composition is generally estimated through surrogate methods. On the other hand, anthropometry is a relatively easy, simple, and inexpensive alternative for estimating adiposity [18, 19]. Adequate training and correct technique are essential for improving the accuracy when performing anthropometric measurements [20].

Different factors have been reported to predict FM in infants, such as sex, age, weight, length, circumferences, and skinfolds [8, 21–23]. Weight-for-length and body-mass index (BMI) are commonly used measures of body proportionality [24, 25]. BMI has shown high correlations with adiposity in adults [26] and moderate correlations in infants [25, 27]; however, is a limited predictor of adiposity in infancy as it does not reflect the amount or distribution of FM [25, 28, 29]. Skinfolds measure subcutaneous fat and have been used predominantly in adults to estimate total body fat. Some FM prediction equations in infants include skinfold measures [8, 21, 22].

Several prediction equations for estimating FM have been developed for neonates [21, 22, 30–34] and older children (≥8yo) [35–39], leaving a gap for infants in the first months of age. Some of these equations used in infants during the first year of life have shown poor agreement [3, 8, 40] with the reference method (DXA, ADP), probably due to age and ethnic differences in the group where equations were derived [3, 8]. Lingwood [41] and Schmelzle [8] developed prediction equations for 0 to 4-month-old Caucasian infants. Schmelzle’s prediction equation shows acceptable validation based on the sum of 4 skinfolds (triceps, biceps, suprailiac, subscapular) and length. Linwood’s FM equation requires the result of a prediction equation for FFM (from bioelectrical impedance analysis) in addition to weight, sex, and length. They reported wide limits of agreement and a 19–21% error in FM estimation. There are no specific equations derived from Hispanic/Mexican infants or a validated one for this population. Considering the relevance of FM in metabolic health, practical and valid body composition methods are needed in early infancy to facilitate the inclusion of FM measurements in clinical practice.

This study aims to develop FM prediction equations using anthropometric and clinical data from Mexican infants at 1, 3, and 6 months (1 M, 3 M, 6 M) of age and to validate them using ADP as the reference method.

## Materials/Subjects and methods

The OBESO (**O**rigen **B**ioquímico y **E**pigenético del **S**obrepeso y la **O**besidad) perinatal cohort is a multidisciplinary study conducted at the Instituto Nacional de Perinatología in Mexico City (2017-ongoing) that aims to determine whether different maternal characteristics (lifestyle, clinical, biochemical, epigenetics) can predict neurodevelopmental and body composition alterations in the child. The cohort characteristics have been described elsewhere [42]. The Ethics and Research Internal Review Board (Project No.3300-11402-01-575-17) approved the study. Participation was voluntary, and all participants signed informed consent.

### Subjects

For this secondary analysis, we included healthy-term newborns (≥37 weeks of gestation) born from healthy adult women (≥18 years, without diseases before pregnancy) with complete anthropometric and FM data. Newborns with postnatal and/or congenital diseases or born from mothers with adverse maternal outcomes (gestational diabetes or preeclampsia) were excluded. Anthropometric and FM assessments were carried out on the same day at 1 M (*n* = 133), 3 M (*n* = 105), and 6 M (*n* = 101). Infants’ clinical data were obtained from the cohort records. Gestational age at birth was estimated based on the ultrasound in the first trimester of pregnancy.

### FM measurement (ADP)

The PEAPOD device (COSMED Inc. USA, California, USA) was used to estimate infant FM (kilograms—kgFM) at 1 M, 3 M, and 6 M. The device was calibrated before each test according to the manufacturer’s protocol. FM measurement started by placing the infant (without clothes and with a cap on the head) on the integrated scale for weight measurement. Then, the infant was placed inside the chamber to measure the body volume. Body density was computed using weight and body volume measurements. Finally, the PEAPOD’s software computed the infant’s FM based on Fomon’s equation (43). We excluded all infants’ FM values <5% [22] from the analysis, due to the fact that it is below the minimum reported values [43–46].

### Anthropometric measurements

Two experienced and trained nutrition professionals performed all anthropometric measurements in duplicate, following Lohman’s methodology [47] and computed the average. Infants were measured at birth (24–72 h), 1 M, 3 M, and 6 M. Birth weight were recorded to the nearest gram using a Baby/Mommy 1582 pediatric scale (Tanita, Tokyo, Japan). The length was measured by two professionals to the nearest millimeter using a SECA 207 infantometer (SECA, Hamburg, Germany). The correct placement of the infant in the Frankfort plane was assured, and the head’s crown and the foot’s heel were in contact with the infantometer. BMI (weight/length^2^) was computed. Nutrition status at birth was classified according to WHO criteria [48].

Circumferences of the head, waist, left arm, and left leg were measured to the nearest millimeter using a W606PM Lufkin tape (Apex Tool Group, Maryland, USA), with the infant in a supine position, except for the head (seated position). Head circumference (HC) was measured at the highest perimeter of the head (maximum point of the occiput and the glabella). The midpoint between the acromion and the olecranon was used as a reference to measure the mid-upper arm circumference (MUAC). Thigh circumference (TC) was measured at the midpoint between the greater trochanter of the femur and the patellar border. At the widest point of the calf, we measured the calf circumference (CC). Waist circumference (WC) was measured at the level of the umbilicus after exhaling.

Skinfolds (biceps –BSF–, triceps –TSF–, subscapular –SSF–, waist-WSF-, thigh –ThSF– and calf –CSF–) were measured using a Lange caliper (Beta Technology, California, USA) holding the skin (one centimeter above the measurement site) between the index finger and thumb. BSF and TSF were measured at the midpoint of the arm, grasping the skin parallel to the long bone. SSF was taken in a diagonal fold (45°) just below the scapula’s inferior angle in the skin’s natural cleavage lines. ThSF and CSF were measured taking the same reference as its circumferences, holding the skinfold parallel to the long bone. A difference >2 mm between both measurements required a third measurement.

### Statistical analysis

Models for FM estimation were developed at 1 M, 3 M, and 6 M in 3 different steps:

1. Variable Selection. Analysis of variable selection considered all variables: infant sex, gestational age at birth, weight, length, circumferences (head, waist, arm, thigh, calf), and skinfolds (biceps, triceps, subscapular, waist, thigh, calf). Data were divided into quintiles to guarantee homogeneity. Eighty percent of data were randomly extracted (Fig. 1a) from each quintile to compose the training set. The Least Absolute Shrinkage Selector Operator (LASSO) was used to select relevant variables. This method identifies non-relevant variables in FM prediction by assigning their betas a zero value. Due to the high variability in the LASSO training, a series of repetitions were performed (Fig. 1b) and the beta values were saved (Fig. 1c). This process was done in 100 blocks of 12 repetitions each. Considering 1200 betas for each variable, those different from zero were selected (Fig. 1d). Relevant variables for the analysis were those that did not get a zero value in at least 500 beta values (Fig. 1e).

**Fig. 1:**
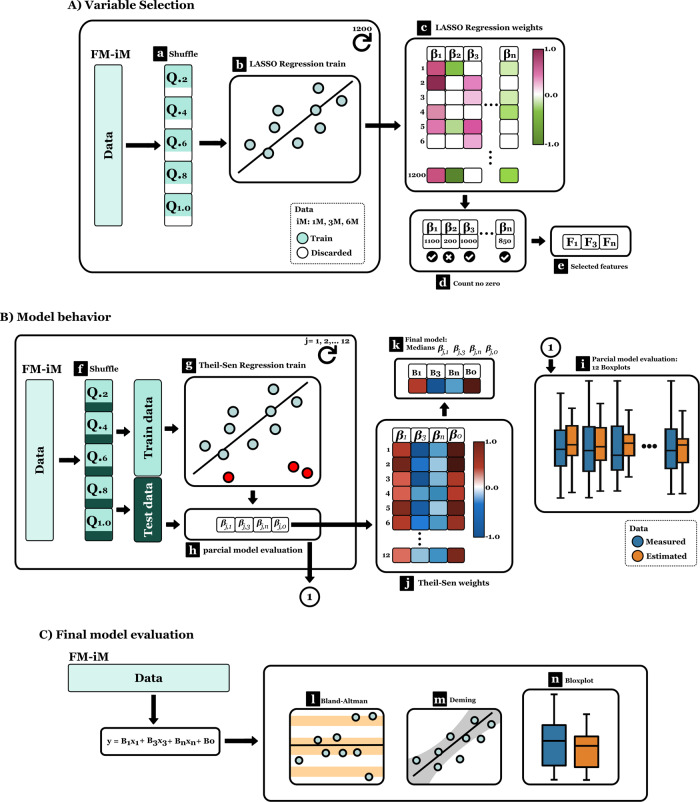
Statistical analysis diagram showing the three-step methodology applied. **A** Variable selection, **B** Model behavior evaluation, **C** Final model evaluation.
2. Model behavior evaluation: A 12-fold cross-validation was performed to evaluate FM prediction in different scenarios. The same quintile strategy was used to train (70%) and test (30%) data. Models were trained using Theil-Sen regressions (Fig. 1g). A partial model was created in each fold (Fig. 1h). Test data and partial models were used to predict FM. Differences were contrasted using boxplot diagrams (Fig. 1i). Partial models were saved, and a final model was calculated based on the median of partial betas from each variable (Fig. 1j, k).
3. Final model evaluation. Bland-Altman plots were generated to evaluate the concordance between predicted and measured FM (Fig. 1l). Deming regressions were used to assess the correlation between estimated and measured values; significant results generally are considered when the confidence intervals from the slope and the intercept contain 1 and 0, respectively, and the p-value from each one (One sample test against true mean value) is >0.05 (Fig. 1m). Boxplots were constructed, and the mean differences between predicted and measured FM were evaluated (Student’s *t*-test/U-Mann Whitney test) (Fig. 1n).

All data processing, model training, and validation was done with Python (v3.9, Python Software Foundation, USA) and scikit-learn (v1.1.1 Scikit-Learn Consortium at Inria Foundation). The software R was used to perform Bland-Altman (R v0.5.1 CRAN.r-project, Austria), Deming regression (R v1.4 CRAN.r-project, Austria), and statistical comparisons (R v4.2.1 R Foundation, Austria).

## Results

From 348 participants in the OBESO cohort, we obtained data from 292 newborns (40 fetal losses, 16 lost to follow-up). We did not include data from 120 healthy infants (55 without specific data, 65 never returned for follow-up) and 33 preterm newborns. FM values < 5% from 7 infants were excluded from the analysis (1 M: *n* = 6, 3 M: *n* = 1, 6 M: *n* = 0). The final study sample for each visit included 133 infants (67 girls) at 1 M, 105 infants (51 girls) at 3 M, and 101 infants (53 girls) at 6 M. The mean (SD) gestational age at birth was 39.00 (1.06) weeks; for girls and boys, the mean (SD) birthweight was 2.90 (0.30) and 3.00 (0.36) kg, and length was 47.24 (1.67) and 47.78 (1.81) cm, respectively. Stunting was observed in 19.7% of newborns, 5.2% were small for gestational age, and no wasting was detected. No differences were observed by sex.

### Anthropometric and body composition data

Table 1 shows infants’ descriptive anthropometric and FM data at 1 M, 3 M, and 6 M. The infants’ mean (SD) age in days at each visit was 35.18 (6.54), 93.71 (7.27), and 185.40 (6.59), respectively. No sex differences in anthropometric data were observed, except for boys who had higher weight, length, and HC in all study periods. MUAC was higher in boys at 6 M (Table 1). Regarding body composition, FM doubled from 1 M to 3 M and increased 33% from 3 M to 6 M. FM was similar between girls and boys at each visit (Table 1).

**Table 1:** Anthropometric and body composition data of infants.

Measurement	All	Girls	Boys	All	Girls	Boys	All	Girls	Boys
	1 month of age			3 months of age			6 months of age		
	*n* = 133	*n* = 67	*n* = 66	*n* = 105	*n* = 51	*n* = 54	*n* = 101	*n* = 53	*n* = 48
Weight (kg)	4.08 (0.51)	**3.97 (0.50)**	4.20 (0.50)*	5.93 (0.74)	**5.68 (0.67)**	6.16 (0.74)*	7.29 (0.84)	**7.02 (0.74)**	7.58 (0.83)*
Length (cm)	52.43 (1.87)	**52.01 (1.78)**	52.86 (1.87)*	59.17 (2.19)	**58.31 (1.71)**	59.99 (2.30)**	65.02 (2.16)	**64.31 (2.06)**	65.79 (2.01)**
Body mass index (kg/m^2^)	14.83 (1.36)	14.66 (1.33)	15.00 (1.38)	16.92 (1.66)	16.71 (1.63)	17.11 (1.67)	17.22 (1.48)	16.96 (1.40)	17.49 (1.51)
Head circumference (cm)	36.74 (1.15)	**36.68 (1.16)**	37.10 (1.02)**	39.93 (1.26)	**39.35 (1.21)**	40.48 (1.06)**	42.52 (1.36)	**41.99 (1.36)**	43.09 (1.11)**
Mid-upper arm circumference (cm)	11.07 (1.00)	10.93 (0.96)	11.20 (1.03)	13.12 (1.04)	12.92 (0.98)	13.30 (1.07)	13.94 (1.09)	**13.68 (0.94)**	14.28 (1.14)*
Waist circumference (cm)	34.41 (2.27)	34.41 (2.13)	34.41 (2.43)	39.28 (2.64)	38.86 (2.35)	39.67 (2.85)	40.96 (2.35)	40.52 (2.14)	41.44 (2.48)
Thigh circumference (cm)	16.85 (1.55)	16.85 (1.46)	16.85 (1.64)	21.41 (1.69)	21.27 (1.78)	21.53 (1.62)	23.75 (1.97)	23.43 (1.92)	24.08 (1.97)
Calf circumference (cm)	12.66 (1.10)	12.60 (0.98)	12.72 (1.21)	15.81 (1.18)	15.61 (1.14)	16.00 (1.20)	17.37 (1.40)	17.22 (1.30)	17.52 (1.49)
Biceps skinfold (mm)	3.67 (1.09)	3.56 (1.06)	3.78 (1.12)	4.81 (1.34)	4.66 (1.24)	4.96 (1.42)	5.19 (1.38)	5.17 (1.45)	5.22 (1.31)
Triceps skinfold (mm)	6.27 (1.55)	6.26 (1.49)	6.29 (1.62)	8.51 (1.98)	8.56 (2.11)	8.45 (1.87)	8.77 (2.00)	8.99 (2.19)	8.53 (1.76)
Subscapular skinfold (mm)	6.27 (1.68)	6.39 (1.62)	6.15 (1.75)	8.16 (2.18)	8.13 (1.88)	8.18 (2.45)	7.69 (1.95)	7.94 (1.89)	7.41 (1.99)
Waist skinfold (mm)	4.94 (1.50)	5.06 (1.50)	4.81 (1.51)	8.00 (2.33)	8.02 (2.20)	7.98 (2.47)	7.99 (2.14)	8.01 (2.07)	7.95 (2.23)
Thigh skinfold (mm)	9.13 (2.38)	9.17 (2.32)	9.10 (2.46)	14.90 (3.19)	14.96 (3.17)	14.84 (3.24)	17.29 (3.18)	17.41 (2.96)	17.16 (3.44)
Calf skinfold (mm)	8.38 (1.90)	8.60 (2.03)	8.16 (1.75)	12.66 (2.43)	12.77 (2.27)	12.56 (2.59)	14.40 (2.48)	14.47 (2.25)	14.33 (2.73)
Fat mass (%)	16.35 (19.15)	18.42 (26.58)	14.25 (4.29)	1.32 (0.48)	1.31 (0.47)	1.33 (0.50)	1.76 (0.58)	1.79 (0.55)	1.73 (0.60)

Anthropometric data expressed as mean (SD).

Student’s t-test: *p < 0.01, **p < 0.001.

Bold values indicates statistical significant P values

### FM prediction equations

**Fig. 2:**
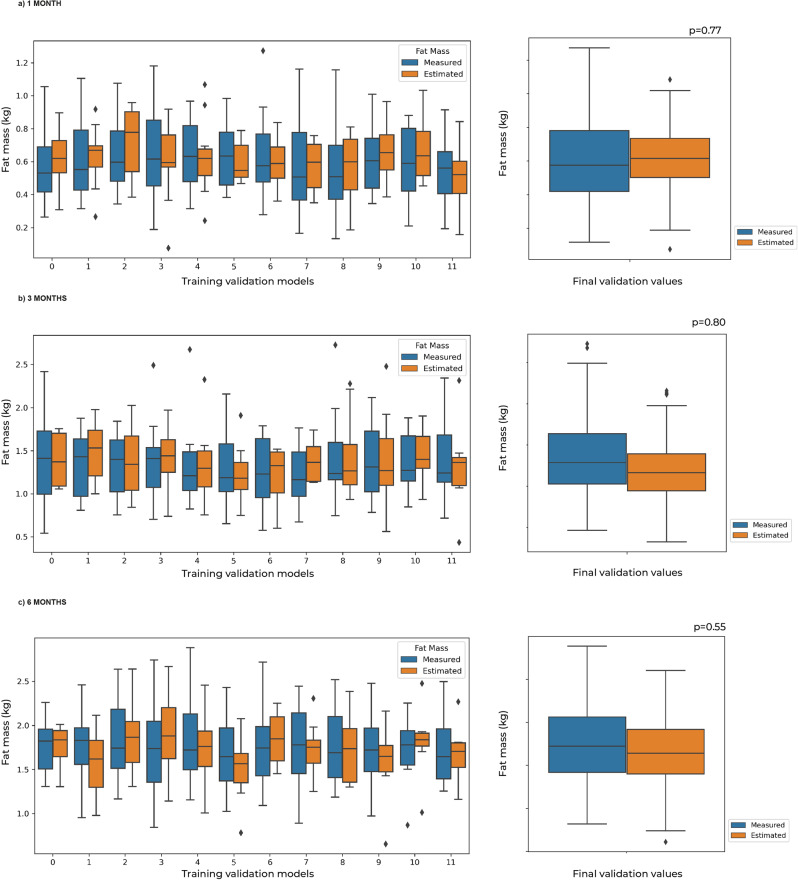
Measured fat mass (kg) and predicted fat mass (kg). At **a** 1 month, **b** 3 months, and **c** 6 months according to Theil–Sen regressions. In each time period the training validation models (left) and final median value from these models (right) are shown.

**Fig. 3:**
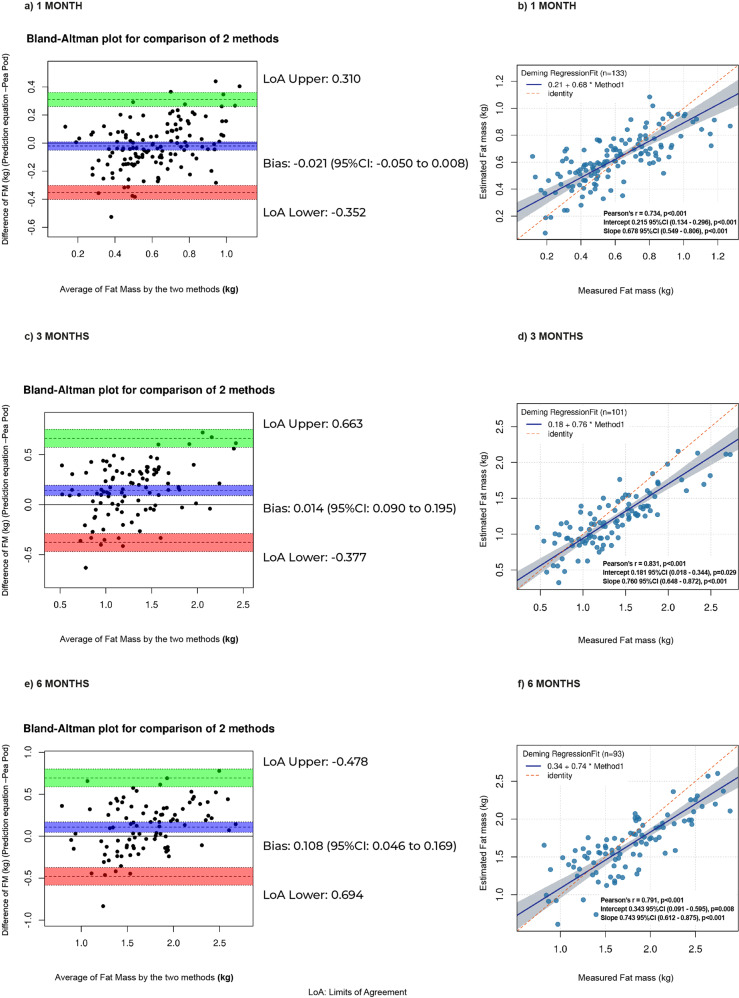
Bland–Altman plots and Deming regressions for comparison of predicted vs measured fat mass (kg). At **a**, **b** 1 month, **c**, **d** 3 months, and **e**, **f** 6 months. Limits of agreement and bias are shown for each Bland-Altman are shown (**a**, **c**, **e**). Correlation coefficient (r), the intercept and slope are presented for each Deming regression graph (**b**, **d**, **f**).

The equation at 1 M included TC, BMI, WSF, ThSF, and SSF (Table 2). No differences in predicted (0.62 kg) and measured FM (0.6 kg) were observed (*p* = 0.77) (Fig. 2a). Predicted values were concordant (Bias: −0.021, 95%CI: −0.050 to 0.008; Limits of Agreement (LoA): Lower: −0.352, 95%CI: −0.401 to −0.302; Upper: 0.310, 95%CI: 0.260 to 0.359). Predicted values outside of LoA were 3.01% for the lower and upper limits (Fig. 3a). Predicted FM showed a high correlation with measured FM (r = 0.734, *p* < 0.001) and an R^2^ of 0.54; intercept and slope in Deming were close to contain 0 and 1 respectively, but did not reach equivalence between methods (*p* < 0.001) (Fig. 3b).

**Table 2:** Proposed equations for estimating fat mass (kg) in infants at 1, 3, and 6 months of age, using air displacement plethysmography as the reference method.

Age	Prediction Equation
1 month	FM (kg) = 0.068(TC) + 0.018(BMI) + 0.026(WSF) + 0.01(ThSF) + 0.009(SSF) − 1.082
3 months	FM (kg) = 0.006(WC) + 0.074(TC) + 0.078(CC) + 0.062(BMI) + 0.024(CSF) + 0.054(SSF) − 0.062(TSF) − 0.053(gestational age at birth) − 1.045
6 months	FM (kg) = 0.030(TC) + 0.163(CC) + 0.023(WSF) + 0.034(ThSF) + 0.019(SSF) − 0.050(gestational age at birth) − 0.858

*FM* Fat mass, *BMI* Body mass index in cm/m^2^, *WC* Waist circumference in cm, *TC* Thigh circumference in cm, *CC* Calf circumference in cm, *TSF* Triceps skinfold in mm, *SSF* Subscapular skinfold in mm, *WSF* Waist skinfold in mm, *ThSF* Thigh skinfold in mm, *CSF* Calf skinfold in mm, Gestational age at birth: in weeks.

The equation at 3 M included WC, TC, CC, BMI, ThSF, SSF, TSF, and gestational age at birth (Table 2). No significant differences were observed between predicted and measured FM (kgFM: 1.2 kg vs 1.35 kg, respectively, *p* = 0.80) (Fig. 2b). The observed bias for this model was 0.014 (95%CI: 0.090 to 0.195) and LoA were: Lower: −0.377 (95%CI: −0.466 to −0.287), Upper: 0.663 (95%CI: 0.573 to 0.752). The percentage of results outside of LoA was 2.97% for lower and 1.98% for upper limits (Fig. 3c). Deming regression presented a high correlation value (*r* = 0.831, *p* < 0.001) and an R^2^ of 0.69; where the intercept (*p* = 0.029) and slope (*p* < 0.001) did not demonstrate equivalence between methods (*p* < 0.001) (Fig. 3d).

The 6 M prediction equation included TC, CC, WSF, ThSF, SSF, and gestational age at birth (Table 2). Predicted vs measured values of FM were 1.65 kg vs 1.76 kg, respectively, showing no statistical difference (*p* = 0.55) (Fig. 2c). In this model the bias was 0.108 (95%CI: 0.046 to 0.169) and estimated LoA values were: Lower: −0.478 (95%CI: −0.584 to −0.373), and Upper: 0.694 (95%CI: 0.589 to 0.800). The percentage of results outside of LoA was 1.08% (from lower and upper limits) (Fig. 3e). The intercept and slope in Deming regression did not contain 0 and 1, respectively (*p* = 0.008 and *p* < 0.001, respectively), but showed a high correlation value (*r* = 0.791, *p* < 0.001) and an R^2^ of 0.63 (Fig. 3f).

## Discussion

In this work, we present anthropometry-based FM prediction equations in infants for the first 6 months of life. FM predicted with our equations showed high correlation values (*r* ≥ 0.73) with FM measured by Pea-Pod at 1 M, 3 M, and 6 M. Our models explained approximately 60% of FM variability at each period. This moderate determination coefficient may be related to the wide distribution of FM data in this group of infants; other published equations had shown moderate to high values [21, 22, 31, 33, 34]. We found significant bias at 3 M and 6 M, which represents a mean sub-estimation of 11.9% (95%CI: 7.5–16.2%) at 3 M and 6.54% (95%CI: 2.78–10.24%) at 6 M. It is not clear what would be an acceptable variation, without affecting their use in clinical practice. Bland-Altman analysis showed that at 3 M, 1.98% and 2.9% of infants were above or below the LoA, respectively. At 6 M, only 1.08% of infants were above and below LoA.

We decided to develop and validate our prediction formulas for Mexican infants in their first 6 months of life instead of using previously validated equations in other populations with different ages. Recent studies have shown different growth and body composition patterns among ethnicities [49]. Prediction equations have been developed for Caucasian [34], Asian [22], and German infants [8]; others have included multiethnic samples [21, 33]. Likewise, it is essential to consider the infant’s age when estimating FM. Slaughter’s equation was developed in children (8–18 years old) and validated in infants (0–4 M), showing significant bias, wide LoA, and high error in the estimation of FM [41]. Schmelzle evaluated in infants (0–4 months) the performance of five FM prediction equations developed in children (8–16 years old), which resulted in a weak correlation and a considerable systematic error overestimating FM [8].

Including different anthropometric measurements from diverse body regions in the equations improves the prediction of FM [29, 50, 51]. WC, ThSF, and CSF may improve the LoA with the reference methods when estimating FM percentage in infants (6–24 months). Many equations based their prediction on weight, length, and/or BMI [31, 33, 41, 52] and a few additional measurements. In two of the developed equations (1 M and 3 M), BMI was a predictive variable. FM prediction models for infants commonly include weight, length, circumferences and/or skinfolds (triceps, subscapular, flank, thigh). De Bruin [23] found that the best combination of anthropometric measurements to predict FM in infants were calf circumference, weight, and the sum of skinfolds (biceps, triceps, subscapular, supra iliac, quadriceps). A strength of our prediction equations is that they included total body mass indices, body circumferences, and skinfolds from the trunk area and the extremities.

The measurement of skinfolds is a well-established and more accessible method for assessing subcutaneous fat [53]. Skinfolds have shown a high degree of agreement and correlation (R^2^ = 0.948) with FM in term infants at birth and 2 and 4 months of age [8]. Josefson [32] found that adding one skinfold measure to weight and length improved the accuracy of estimating neonatal FM. Aris and cols [22] reported that adding SSF to the equation improved the prediction of neonatal FM. Most FM prediction models include at least one skinfold measurement, even for infants [8, 21]. Other FM estimation equations, such as Huvanandana’s [31] did not include skinfolds and reported lower R^2^ values. Skinfold measurement could be challenging in infants during the first months of life for several reasons, such as small body size, infants not staying still, and sensitive skin, among others. Likewise, skinfold measurements are prone to a high degree of error; hydration status, the personnel’s experience, the technique, and equipment used (caliper, site of measurement, time holding the skin) influence their measurement, affecting validity and reproducibility [54].

Our models included different body circumferences, which are easier to measure and more reproducible than skinfolds [23, 55]. Girth parameters are important as the human body is theoretically divided by cylinders [55] and have been used to establish theoretical models to predict body composition [33, 56]. WC measures central fatness and has been associated with metabolic alterations and cardiovascular risk. CC was the best single predictor of total body fat (R^2^ = 0.83) in the De Bruin analysis [23]. Daly-Wolfe [57] found that TC accounted for 63.0% of the variability of FM by ADP (*p* < 0.001) in term infants. Heymsfield hypothesizes that combining individual or multiple site circumference measurements with BMI may provide additional body composition information by constructing an individual’s “somatogram” [56].

FM is associated with different health outcomes throughout the life cycle [58]. Therefore, body composition assessment and adequate body fat classification are crucial. Considering the high accretion observed in this period [41, 59, 60], more studies are needed to define optimal FM in infants. Exclusive breastfeeding has been associated with higher FM, probably representing a programming factor in preventing obesity [60].

The strengths of our study are that we measured FM using ADP, a validated method to assess body composition in different populations of infants [14–17]. The outcome variable was absolute (kg) rather than relative (%) FM, which is a better indicator of body composition in anthropometry [22, 41]. The inter-observer coefficient values between both professionals were minimal. Equations for a specific month of life are advantageous because of accelerated growth and differences in FM accretion during the first six months [41, 59, 60]. The 12-run cross-validation allowed us to evaluate the variability of Theil-Sen regression estimations against different train/prediction groups instead of using only one validation group.

We recognize some limitations in our study. The relatively small sample size could restrict the prediction power of the models. Our models included many measurements, which can be challenging for clinicians, where some measurements, especially skinfolds, require trained personnel, adequate equipment, and standardized methodology.

## Conclusion

Nutritional assessment should include body composition since early infancy because it could affect the programming of adiposity later in life. Estimating FM with anthropometry-based prediction equations is a more accesible and inexpensive alternative but should be validated for the population where they will be used. These equations are a valuable tool in research or in clinical settings for evaluating body fat in a similar population of infants, where more precise and reliable body composition methods are unavailable. Further research should focus on establishing FM cut-off points to define metabolic risk.

## Data Availability

The datasets generated and/or analyzed during the current study are available from the corresponding author on reasonable request.
